# Effect and process evaluation of a real-world school garden program on vegetable consumption and its determinants in primary schoolchildren

**DOI:** 10.1371/journal.pone.0214320

**Published:** 2019-03-21

**Authors:** Nele Huys, Greet Cardon, Marieke De Craemer, Ninotchka Hermans, Siska Renard, Marleen Roesbeke, Wout Stevens, Sara De Lepeleere, Benedicte Deforche

**Affiliations:** 1 Department of Movement and Sport Sciences, Faculty of Medicine and Health Sciences, Ghent University, Ghent, Belgium; 2 Department of Public Health and Primary Care, Faculty of Medicine and Health Sciences, Ghent University, Ghent, Belgium; 3 Logo Gezond+, Ghent, Belgium; University of British Columbia, CANADA

## Abstract

**Objectives:**

This study aimed to investigate the effectiveness of a school garden program on children’s vegetable consumption and determinants and to gain insight into the process of the program.

**Methods:**

The “Taste Garden” is a real-world nine-week school garden program developed and implemented by a local organization. A total of 350 children (149 intervention group, 201 control group) filled out questionnaires on vegetable consumption, determinants and process of the program. Additionally, teachers filled out a process evaluation questionnaire. For effect evaluation, interaction effects (time x group) were considered, using multilevel repeated measures analyses in MLwiN 3.02. Interaction effects were repeated, taking into account quality of implementation (time x implementation group). Process evaluation was descriptively assessed with SPSS 24.0.

**Results:**

Overall, beside some practical concerns of teachers, the program was well perceived by teachers and children. However, an intervention effect of “The Taste Garden” was only found for knowledge (p = 0.02), with a very small effect size (0.55%). When taking into account implementation quality, only small effects were found for awareness (p between 0.005 and 0.007 and an effect size of 0.63%) and knowledge (p between 0.04 and 0.09 and an effect size of 0.65%).

**Cconclusions:**

Evaluation of the real-world “Taste Garden” program, which was positively perceived by teachers, showed no effects on vegetable consumption and small effects on its determinants. Adaptations of the current format and longer follow-up periods are therefore recommended.

## Introduction

Evidence shows that a sufficient intake of vegetables is related to a decreased risk of all-cause mortality and the development of several non-communicable diseases [1–3]. Childhood is seen as an important period for the development of healthy eating behaviours, including vegetable consumption [4, 5]. Furthermore, healthy behaviours adapted in childhood track into adulthood [6, 7]. For the prevention of chronic diseases, the World Health Organization (WHO) recommends to consume ≥ 400 grams fruit and vegetables [8]. Recent data from 44 countries in Europe and North America indicated however that only 39% of 11-year olds consume vegetables on a daily basis [9]. Mean vegetable intake per day in the European PRO GREENS sample ranged from 73 to 141 grams per day [10]. These low vegetable intake numbers underline a clear need for effective programs promoting vegetable consumption in children.

Schools are an appropriate setting for the implementation of programs promoting vegetable intake among children for two main reasons. First, the majority of children can be reached through schools [11] (e.g. in Flanders (Belgium) 98% of children between 6 and 12 years old are registered in school [12]). Second, schools provide the opportunity for continuous, intensive contact with children [13]. According to a review of Van Cauwenberghe et al. (2010), school-based interventions focusing on the promotion of healthy eating including only an educational component (i.e. classroom-based activities) or only an environmental component (i.e. fruit and vegetable subscription or distribution) have had limited effect on increasing children’s fruit and vegetable consumption [14]. In contrast, multicomponent interventions (including both educational and environmental components) show more promising results in increasing children’s fruit and vegetable consumption [14–16]. Although school-based interventions mostly promote both fruit and vegetable consumption, previous research showed that multicomponent school-based interventions are more effective in changing fruit consumption than in increasing vegetable consumption [15]. Therefore, it seems important to develop interventions that focus specifically on promoting vegetable consumption in schoolchildren. Blanchette et al. (2005) showed that such interventions should aim to increase availability and accessibility of vegetables and improve children’s taste preferences for vegetables, as these are most strongly related to consumption [16]. An approach that incorporates both the environment and education and that takes into account the intervention aims outlined by Blanchette et al. (2005) is the experiential learning approach. Research showed that experiential learning approaches are more effective in influencing children’s vegetable consumption than other school-based interventions (e.g. nutrition education) [17, 18]. A specific experiential learning approach is setting up school gardens, which are defined by the Food and Agricultural Organization of the United Nations as “cultivated areas around or near to primary schools, which can be used mainly for learning purposes but could also generate some food and income for the school” [19]. Emerging quantitative evidence in the review of Ohly et al. (2016) showed that school garden programs, developed and/or led by a research team (e.g. the Stephanie Alexander Kitchen garden project in Australia and the Royal Horticultural Society Campaign for School gardening in London) had positive effects on children’s willingness to taste vegetables, preferences for vegetables and knowledge and attitudes toward the consumption of vegetables [20]. As the results of the quantitative evidence in the study of Ohly et al. (2016) [20] seem promising, a Flemish (Belgian) local health promotion service (Logo Gezond+) developed a school garden program of nine weeks, named “The Taste Garden”, to promote vegetable consumption in primary schoolchildren and implemented this project in the region of Ghent (Flanders, Belgium). The first aim of the present study was to explore the effectiveness of the latter real-world school garden program in Flanders (Belgium) on vegetable consumption in primary schoolchildren. It was hypothesized that “The Taste Garden” would have positive effects on vegetable consumption.

Based on the underlying theory of the intervention mapping approach (Bartholomew et al., 2016) and the PRECEED-PROCEED model (Green & Kreuter, 1999), change in behaviour occurs through the change in determinants of this behaviour [21, 22]. Several important psychosocial determinants of vegetable consumption are emerging in the literature, such as knowledge and awareness [16, 23, 24], attitude and preference [16, 23, 25], self-efficacy [16, 23, 24] and social influence [23–25]. Therefore, the second aim of this study was to test the hypothesis that “The Taste Garden” would be effective in changing determinants of vegetable consumption that were addressed within the program (i.e. awareness, knowledge, social influence, self-efficacy and attitudes) in primary schoolchildren.

Although the quantitative evidence in the review of Ohly et al. (2016) is promising [20], none of these studies integrated a process evaluation in their effect evaluation. Nonetheless, it is also important to evaluate the process of the program, as variability in the impact of an intervention may highly depend on the quality of the implementation [26–28]. Saunders, Evans & Joshi (2005) developed a guide for program implementation [29]. Within this guide, they suggest several key elements of process evaluation for public health interventions: fidelity (quality), dose delivered (completeness), dose received (exposure), dose received (satisfaction), reach (participation rate), recruitment and context. Following this guide, the third aim of the present study was to assess whether the implementation of the program was related to differences in intervention effects. The researchers hypothesized that “The Taste Garden” would be more effective in changing (determinants of) vegetable consumption in schools with a higher implementation quality than schools with a lower implementation quality.

## Materials and methods

### Participants

Schools were recruited between June and October 2015. Intervention schools were recruited by Logo Gezond+. All primary schools in Ghent (n = 113) were invited to implement “The Taste Garden”, which was a program free of charge. A total of 87 schools were interested in implementing the program, received the intervention material and were invited to participate in the evaluation of the program. Eventually, a total of 32 schools wanted to participate in the evaluation. After applying exclusion criteria, 13 schools were eligible for participation in the evaluation of the program. Exclusion criteria were (1) already having a school garden and (2) being a school with special education (i.e. education for children who are unable to attend regular education for several reasons, such as disabilities or emotional or behavioural problems). To compile a control group, researchers contacted all schools that were not interested in implementing in “The Taste Garden” or did not plan to implement the program in the current academic year via mail and phone calls. After applying the same exclusion criteria, four control schools were included. As such, this study had a non-equivalent pretest-posttest control group design with intervention and control schools in the region of Ghent.

**Fig 1:**
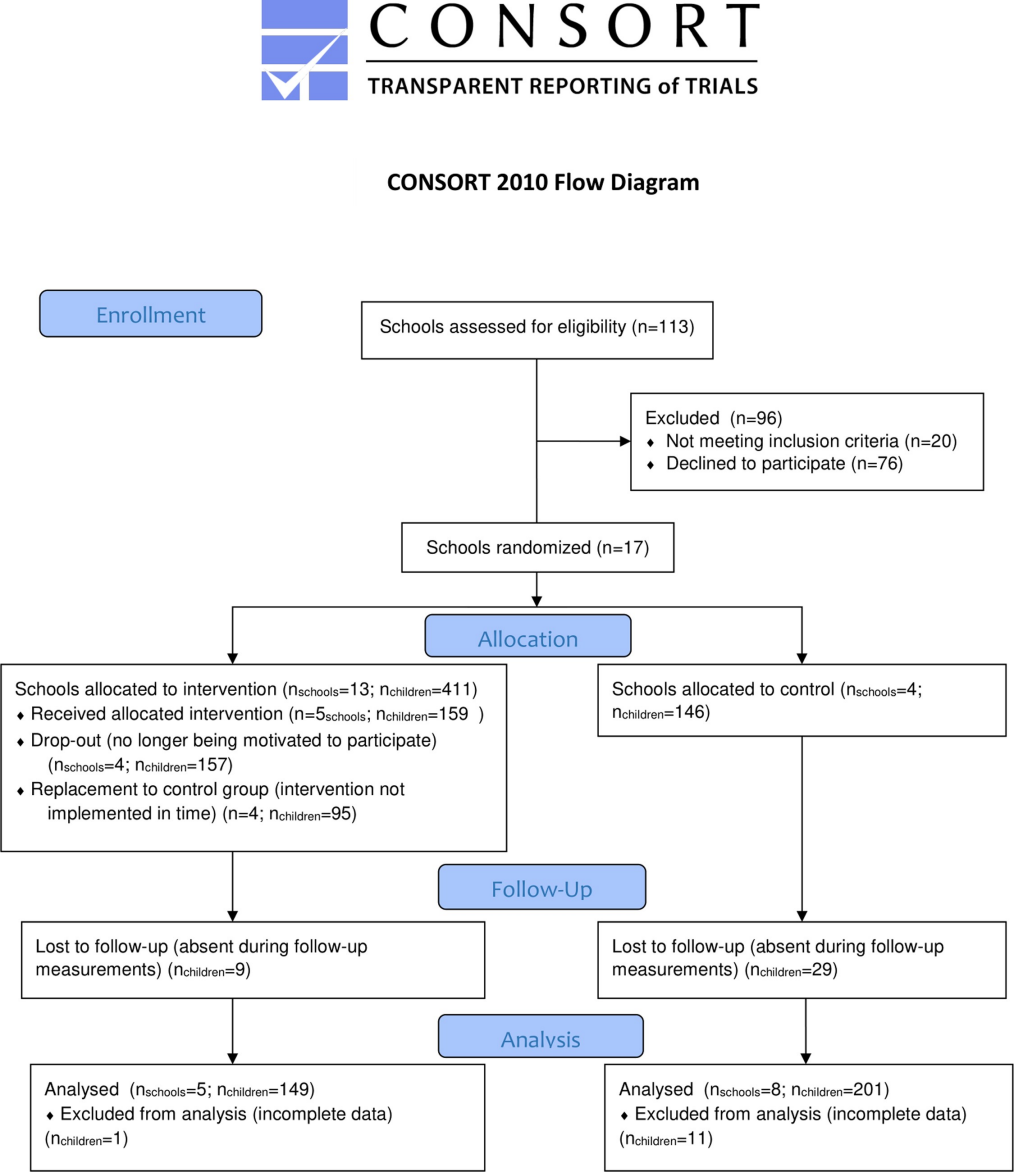
Participant flow through the study for the effect evaluation.

Following the recruitment of schools, one teacher per school was in charge of recruiting children in grades five and six (aged 10–12 years) for the effect evaluation. Children of this age were chosen for the evaluation, as the ability to independently fill out a questionnaire was important for this study. Parental consent was obtained via an opting-out method. One week before baseline measurements, letters were distributed to parents of all children explaining the nature of the study. Parents who did not wish their child to participate in the study had to sign the form and return it to the school. A total of 551 (312 intervention and 239 control) children participated in the study. During the period of the program, three of the intervention schools dropped out of the study due to no longer being motivated to implement “The Taste Garden” or no longer being motivated to participate in the study. Another four intervention schools did not implement the program, because they did not set up the school garden in time. Therefore, these four schools were included in the control group. In schools of the control group, there was a large drop-out of children, as several children changed schools or were absent during the follow-up measurements. Only children who had data on all variables were kept in the sample. Eventually, the sample consisted of 149 children in the intervention group (five schools) and 201 children in the control group (eight schools) (Fig 1).

### Instruments

The evaluation focused on vegetable consumption and its determinants. On both measurement periods (baseline and follow-up), children filled out a questionnaire in the classroom in the presence of two researchers, which took about 30–45 minutes. At follow-up, children and teachers of participating classes of intervention schools were also invited to fill out a process evaluation questionnaire.

#### Effect evaluation questionnaire

The questionnaire used for the effect evaluation was based on the Dutch version of the Pro Children questionnaire. This reliable and valid questionnaire was developed to assess (determinants of) fruit and vegetable intake in 11- to 12-year-old children [30]. As the current study only assessed vegetable consumption, only these questions were kept in the questionnaire. Questions assessing socio-demographics were added.

**Socio-demographic variables.** The questionnaire assessed age (i.e. ‘How old are you?’), sex (‘Are you a girl or a boy?’) and socioeconomic status (SES). The latter was assessed using the validated Family Affluence Scale II (FAS II) as Andersen et al. (2008) showed that children’s reports on the scale has good convergence with parental reports of SES and the scale can be used to rank children according to SES [31]. The scale questions child’s SES in four items [32, 33]: ‘Does your family own a car?’ (no [0]; yes, one [1]; yes, two or more [2]), ‘Do you have your own bedroom?’ (no [0]; yes [1]), ‘How many computers does your family own?’ (none [0]; one [1]; two [2]; more than two [3]) and ‘During the past 12 months, how many times did you travel away on holidays with your family?’ (not at all [0]; once [1]; twice [2]; more than twice [3]). Scores per question were added up to gain an overall FAS-score, with a maximum score of 9. A FAS-score of 0–2 signified a low SES, a score of 3–5 a medium SES and a score of 6–9 a high SES [25].

**Vegetable consumption.** To assess vegetable consumption, the questionnaire contained three questions. First, children were asked how often they usually eat vegetables (raw or cooked) (eight answer options ranging from ‘never’ to ‘each day, more than twice a day’). Second, children were asked how many vegetables they usually eat per day (answer options ranged from ‘none’ to ‘12 tablespoons per day’). To facilitate children to correctly estimate their vegetable consumption, researchers showed pictures of what a number of tablespoons of vegetables looks like on a plate. Third, children were asked how often they usually eat soup (answer options ranged from ‘never’ to ‘more than once a day’).

**Determinants of vegetable consumption.** The questionnaire assessed determinants of vegetable consumption, such as awareness (1 question, i.e. ‘Do you think you eat many or few vegetables?’, 5-point scale ranging from (1) very few to (5) very much), knowledge (1 question, i.e. ‘How many vegetables do you have to eat per day to eat healthy?’, 6-point scale ranging from (0) none to (5) > 12 tablespoons/day), social norm (1 question, i.e. ‘Do you think you eat more or less vegetables than your peers?’, 5-point scale ranging from (1) much less to (5) much more), parental influence (3 questions, e.g. ‘Do your parents demand you to eat vegetables every day?’, 5-point scale ranging from (0) never to (4) always, Cronbach’s Alpha = 0.77), self-efficacy (3 questions, e.g. ‘It is hard for me to eat vegetables every day’, 5-point scale ranging from (1) totally disagree to (5) totally agree, Cronbach’s Alpha = 0.78) and attitude (4 questions, e.g. ‘If I eat vegetables every day, I feel good’, 5-point scale ranging from (1) totally disagree to (5) totally agree, Cronbach’s Alpha = 0.84). Content and answer categories can be found in S1 Table.

#### Process evaluation

Process evaluation data were collected using questionnaires for children and teachers who received or implemented the program. The questionnaires are described below separately for children and for teachers.

**Children.** Three questions assessed to what extent children appreciated the “Taste Garden”-project. Children were asked to what extent they liked working with the garden planters (5-point scale: not fun at all–a lot of fun), if they liked the lessons regarding vegetables and the garden planters (5-point scale: not fun at all–a lot of fun) and how often they wanted to work with the garden planters in the future (5-point scale: never–every day).

**Teachers.** Process evaluation questions regarding implementation quality for teachers are shown in Table 1. A total of 6 teachers, from 4 of the 5 intervention schools filled out the process evaluation questionnaire. None of the teachers of the 5^th^ school completed a process evaluation questionnaire. The questionnaire was based on essential process evaluation components identified by Saunders et al. (2005) (i.e. reach, fidelity, dose delivered, dose received–exposure, dose received–satisfaction, recruitment and context) [29]. The components ‘dose received—exposure’ and ‘reach’ were not assessed as this study was a pilot project. After each question, there was room for teachers to further explain their answers or highlight other important aspects not yet assessed. At the end of the questionnaire, teachers could add some comments and suggestions.

**Table 1:** Process evaluation questions for teachers.

Dose delivered	Satisfaction	Fidelity	Context
1. *How many lessons from the manual did you teach*? • 0 = 0–4 lessons • 1 = 5–9 lessons	Following items were assessed on a 5-point scale • 0 = completely disagree–nor agree/nor disagree • 1 = agree–completely agree 2. *I like the program* 3. *The program was useful* 4. *The program was interesting* 5. *The program was instructive* 6. *The program was motivating* 7. *The program was difficult (negatively coded* Following items were assessed on a 5-point scale • 0 = certainly not–sometimes/sometimes not • 1 = mostly–certainly 8. *The lessons were too short (negatively coded)* 9. *The moments in the school garden were too short (negatively coded)* 10. *The project was adapted to the age of the children* 11. *The manual was clear* The following item was assessed on a 5-point scale • 0 = extremely negative–impartial • 1 = rather positive–extremely positive 12. *What is your opinion on the program*?	13. *Did you have enough garden planters at your disposal*? • 0 = no • 1 = yes Following item was assessed on a 5-point scale • 0 = certainly not–sometimes/sometimes not • 1 = mostly–certainly 14. *Could you teach the lessons as described in the manual*?	Following items were assessed on a 5-point scale • 0 = certainly not–sometimes/sometimes not • 1 = mostly–certainly 15. *To what extent did lack of time prevent you from teaching certain items from the manual*? *(negatively coded)* 16. *To what extent did lack of interest prevent you from teaching certain items from the manual*? *(negatively coded)* 17. *To what extent did difficulties to integrate in the curriculum prevent you from teaching certain items from the manual*? *(negatively coded)* The following item was assessed on a 5-point scale • 0 = completely disagree–nor agree/nor disagree • 1 = agree–completely agree *18. I found the project easy to implement within the usual lessons*

Based on the process evaluation components, an implementation score was computed for each teacher. All essential process evaluation components were dummy coded and added up to a total score of 18. The higher the implementation, the better the quality of implementation of the “Taste Garden”. Based on the individual teachers’ implementation score, a mean implementation score per primary school was calculated. The median implementation score was 12 (min: 7, max: 15). Based on the implementation score, intervention schools were divided into two groups: (1) schools with an implementation score below or equal to the median (2 schools, scores 7 and 12) and (2) schools with an implementation score above the median (2 schools, scores 13 and 15). This resulted in a qualitative variable with three implementation-groups: control group (0), low implementation intervention group (1), high implementation intervention group (2).

In addition, teachers were asked if they saw any benefits of “The Taste Garden” project on the vegetable consumption of children. Teachers were also asked if they would continue using the garden planters and the educational guide in the future.

#### Procedure

The “Taste Garden” was developed by Logo Gezond+, which is an organization in Flanders contributing to the realization of the Flemish health objectives at the local level on behalf of the Flemish government. Logo Gezond+ makes different settings (i.e. local authorities and associations, education, the care sector and companies) familiar with validated prevention methods and materials, encourages them to collaborate and consult with each other, supports them in the execution, coordination and evaluation of actions and signals needs experienced by the Flemish government (https://logogezondplus.be). The idea behind the program was that a school garden is an ideal way to work on healthy food at school and in the classroom in a fun and playful way. The main aim of the program was to increase the vegetable consumption of primary schoolchildren (aged 6 to 12 years) by growing their own vegetables. To reach this goal, children were involved in the process of sowing, taking care of and harvesting vegetables. Participating schools received program materials, which existed of a starter pack for a school garden and an educational guide for teachers. The starter pack contained a garden planter, vegetable mould, sowing seeds and garden material (e.g. shovel, rake, watering can). The educational guide for teachers not only dealt with vegetable consumption, but also with the broader reality of health and healthy food, as the goal of Logo Gezond+ was to provide teachers with tools to work on school health in general. The educational guide existed of two parts. The first part (19 pages) elaborated on the practical side of school gardening in garden planters by giving a step-by-step plan for creating the school garden. The second part of the guide (100 pages) contained suggestions to work on healthy nutrition in the classroom, organized in chapters per two grades, with links to the school garden. The guide for the first and second grade (6–8 year olds) focused on taste development, tasting and discovering fruit and vegetables and was organized in 9 subchapters. The guide for the third and fourth grade (8–10 year olds) focused on teaching knowledge and skills to choose for a healthy meal and was organized in 8 subchapters. The guide for the fifth and sixth grade (10–12 year olds) focused on learning to consciously choose healthy food and was organized in 9 subchapters. Each subchapter approached a specific theme (see Table 2). Classroom activities within the different subchapters were inspired by previous methodologies of Logo Gezond+ for primary schoolchildren, focused on several determinants of health behaviour (as presented in Table 2) and consisted for example of cooking activities, quizzes, stories and assignments. The schools and teachers were responsible for the implementation of the intervention materials (i.e. mounting the school garden and applying the suggestions of the educational guide in the classroom).

**Table 2:** Themes arising in the different chapters of the educational guide per grade.

Theme	Grade 1–2	Grade 3–4	Grade 5–6	Determinants
The active food guide pyramid	X	X		Knowledge, awareness, attitudes
Healthy breakfast	X	X	X	Knowledge, awareness, attitudes
Healthy lunch	X	X	X	Knowledge, social norm, self-efficacy, attitudes
Physical activity	X	X	X	Knowledge
Healthy snacks	X	X	X	Knowledge, social norm, social support, self-efficacy, attitudes
Healthy hot meal	X		X	Self-efficacy, social support
Healthy parties	X	X	X	Knowledge, awareness, attitudes
Healthy treat	X		X	Knowledge, self-efficacy
Foreign vegetables and exotic fruit	X	X	X	Knowledge, attitudes
Water consumption		X		Knowledge, attitudes
Origin of fruit and vegetables			X	Knowledge

Data were collected twice, nine weeks apart. Baseline measurements took place in September/October 2015 and follow-up measurements took place in December 2015. Between the two measurement periods, teachers of intervention schools received the program materials with at least one starter pack for a school garden. They implemented “The Taste Garden” in their school and control schools followed the standard school curriculum. This study was conducted according to the guidelines laid down in the Declaration of Helsinki and all procedures were approved by the Ethics Committee of the University Hospital of Ghent (project 2018/1245, Belgian registration number B670201837631).

### Data analysis

Prior to all analyses, all outcome measures were checked for normal distribution (skewness and kurtosis < 1.00). All outcome measures were normally distributed.

Descriptive statistics (using SPSS 24.0 for Windows) were computed to describe the sample characteristics, process evaluation of children and the additional process evaluation questions for teachers. To assess the effectiveness of “The Taste Garden” program on children’s vegetable consumption and determinants and to control for clustering of primary schoolchildren in schools, multilevel repeated measures analyses were conducted using MLwiN 3.02 (Centre for Multilevel Modelling, University of Bristol, UK) with three levels: time, child and school. To compare the children who were included in the analyses and those who were excluded from analyses, attrition analyses were conducted as a logistic regression with two levels (child and school) in MLwiN 3.02. All analyses were adjusted for sex, age and SES of the child.

The two-way interaction effects of ‘time x group’ were considered for the total sample. To study the effect of the quality of implementation on children’s vegetable consumption and its determinants, the effect evaluation was repeated, integrating the three implementation groups (control group, low implementation intervention group and high implementation intervention group), using repeated measures analyses (time x implementation group) with three levels (time, primary school child and school). For all analyses, statistical significance level was set at p < 0.05. All effects with a p-value between 0.05 and 0.10 were interpreted as a trend towards significance.

To calculate effect sizes, partial eta squared (η_p_^2^) was used. A small effect size is identified by an effect of 0.02 (2% explained variance), a medium effect size by an effect of 0.12–0.25 (12–25% explained variance) and a large effect size by an effect of 0.26 or higher (26% explained variance or higher).

## Results

### Sample characteristics

The mean age of the sample was 10.5 years (± 0.7, range between 9 and 13 years old) and 51.7% were girls. The sample consisted of 2.9% of participants with a low SES, 24.3% with a medium SES and 72.9% with a high SES. The daily vegetable consumption at baseline was 139.1 grams (± 97.0, min: 0 grams, max = 325 grams). There was only a trend towards a significant difference between intervention and control group in SES at baseline, with the intervention group having a lower SES compared to the control group (p = 0.05). Attrition analyses showed no significant differences in age, sex or SES of primary schoolchildren who were excluded from analyses and those who were not excluded (OR_age_ = 1.04; 95% CI_age_ = 0.75, 1.33; OR_sex_ = 1.21; 95% CI_sex_ = 0.95, 1.46; OR_SES_ = 1.08; 95% CI_SES_ = 0.79, 1.36). The sample characteristics can be found in Table 3.

**Table 3:** Sample characteristics.

	Total	Intervention group	Control group	p-value for difference between intervention and control group
Age in years	10.45 (± 0.73)	10.50 (± 0.73)	10.42 (± 0.72)	0.35
Sex in % girls	51.7	51.0	52.2	0.82
SES	2.9% low	5.4% low	1.0% low	0.05
	24.3% medium	24.2% medium	24.4% medium	
	72.9% high	70.5% high	74.6% high	
Daily vegetable consumption in grams	139.63 (± 97.21)	131.24 (± 100.53)	144.93 (± 94.03)	0.19

### Intervention effects

Results are shown in Table 4. There was a significant positive intervention effect for knowledge (β = 0.15, p = 0.02; η_p_^2^ = 0.55%). Knowledge in the intervention group increased, while knowledge in the control group decreased. No other significant intervention effects were found.

**Table 4:** Interaction effects for the intervention outcomes.

	Baseline	Follow-up	Time*Group		
			β (SE)	95% CI	η_p_^2^ (%)
Vegetable consumption					
Control	141.66 g/day	134.34 g/day	1.78 (10.25)	-18.30; 21.86	< 0.01
Intervention	137.46 g/day	131.92 g/day			
Soup consumption					
Control	2.89 times/week	2.98 times/week	0.04 (0.25)	-0.46; 0.53	< 0.01
Intervention	3.00 times/week	3.13 times/week			
Awareness (score /4)					
Control	2.47	2.50	-0.13 (0.09)	-0.38; 0.04	0.13
Intervention	2.41	2.32			
Social norm (score /4)					
Control	2.16	2.18	0.05 (0.09)	-0.27; 0.23	0.02
Intervention	1.94	2.01			
Parental influence (score /4)					
Control	2.37	2.35	-0.02 (0.06)	-0.14; 0.11	< 0.01
Intervention	2.42	2.32			
Knowledge (score /1)					
Control	0.50	0.42	0.15 (0.07)*	0.02; 0.28	0.55
Intervention	0.43	0.50			
Self-efficacy (score /4)					
Control	2.90	2.97	-0.01 (0.10)	-0.21; 0.19	< 0.01
Intervention	2.80	2.86			
Attitude (score /4)					
Control	2.80	2.75	-0.09 (0.09)	-0.26; 0.08	0.07
Intervention	2.82	2.68			

SE = standard error

CI = confidence interval

*p < 0.05

### Intervention effects taking into account implementation score

When revising the intervention effects taking into account implementation scores, significant interaction effects were found for awareness (η_p_^2^ = 0.63%) and knowledge (η_p_^2^ = 0.65%). The change in mean score for awareness was significantly different (β(SE) = -0.30(0.11), p = 0.005) between schools of the control group (score + 0.03) and low implementation intervention schools (score -0.27). Similarly, a significant difference was found (β(SE) = 0.39(0.14), p = 0.007) between low implementation intervention schools and high implementation intervention schools (score + 0.12). No significant difference for awareness was found between the control group and the group with a high implementation score. The change in mean score for knowledge was significantly different (β(SE) = 0.17(0.08), p = 0.04) between schools of the control group (-0.08 units) and low implementation intervention schools (+0.09 units) and a trend towards a significant difference (β(SE) = 0.16(0.10), p = 0.09) was found between control schools and high implementation intervention schools (+0.08 units). There was no significant difference for knowledge between low implementation intervention schools and high implementation intervention schools.

### Process evaluation of children

On average, children liked to work in the school garden (mean score on 5: 3.95 (± 1.23)), however, 10 children mentioned not to like it (10.1%). Most children also liked the lessons (mean score on 5: 3.39 (± 1.25)), however 19 children did not like the lessons (17.9%). In the future, children would like to work more in the school garden (mean score on 5: 3.67 (± 1.25)), however, 17 children indicated they wanted to work less or never wanted to work in the school garden again (16.5%).

### Process evaluation of teachers

In total, six teachers (out of four schools) filled out the process evaluation questionnaire. On average, teachers implemented four of the nine lessons. Lack of time prevented them to teach certain items of the educational guide (n = 6). The majority of teachers (n = 4) mentioned they could not implement the lessons as described in the manual and found it difficult to implement the themes of the educational guide in their normal lessons (n = 4).

Some teachers (n = 2) did not see benefits of “The Taste Garden” project on children’s vegetable consumption, while others (n = 2) did see benefits. Two teachers cited the short duration of the intervention (i.e. nine weeks) as a reason for the lack of benefits. Furthermore, one teacher wrote down that the timing of the project (i.e. fall) was wrong. All teachers (n = 6) were planning to continue using the garden planters in the future and five teachers were planning to continue using the educational guide. However, one teacher indicated he/she probably will not continue using the educational guide.

## Discussion

The present study examined the effectiveness of “The Taste Garden” program on children’s vegetable consumption and its determinants and the influence of the quality of implementation of the program on the effectiveness. Although the systematic review and meta-analysis of Dudley et al. [17] and the meta-analysis of Langellotto & Gupta [18] indicated that experiential learning approaches are more effective in influencing children’s vegetable consumption and related determinants than other school-based interventions (e.g. nutrition education), the findings of the current study indicated no effects on vegetable consumption and soup consumption. This lack of effects on the consumption has also been observed in previous school garden programs, as mentioned in the review of Ohly et al. [20]. In addition, the current study found only a small (effect size < 1%) significant positive intervention effect on knowledge regarding recommendations for vegetable consumption. When taking quality of implementation into account, the current study showed only a significant difference between intervention schools with a low implementation score and a high implementation score for awareness, with awareness decreasing in the low implementation group and increasing in the high implementation group. This result suggests that quality of implementation played a role in effectiveness of the “Taste Garden” intervention. Schools that, on average, delivered more of the intervention content, had a better satisfaction, a greater fidelity and less contextual barriers to implement the intervention having higher odds of changing children’s determinants of vegetable consumption. This is in line with other school-based interventions promoting fruit and vegetable consumption in children [26–28]. Nevertheless, the effect on awareness in the current study was also very small and may not be biologically relevant. Our limited findings regarding determinants were in contrast with school gardening research. Both the review of Ohly et al. [20] and the study of Somerset and Markwell [34] found evidence for significant positive intervention effects of school garden programs on different determinants under investigation in the current study (i.e. awareness, modelling, knowledge, self-efficacy and attitudes).

The lack of effects of the “Taste Garden”-project could be attributed to the fact that the project was a real-world program, which means it was not developed, nor led by a research team. Evidence states that linking theory, research and practice is found to increase the effectiveness of nutrition education programs [35]. Although the “Taste Garden” was evidence-based, based on previous evidence-based projects of Logo Gezond+ and targeted several determinants, results did not show many significant effects. It may be possible that effectiveness could have been increased when the project was led by social cognitive theory, as a majority of the school garden projects in the review of Ohly et al. [20] shown to effectively increase vegetable intake, food preferences or knowledge and attitudes towards food, used the social cognitive theory to develop the intervention, such as the ‘How do you grow? How does your garden grow?’-program of Morgan et al. [36, 37] and the garden-enhanced nutrition education curriculum of Morris et al. [38, 39]. This theory is seen to be most effective when aiming to change health-related behaviours in schoolchildren [40].

Furthermore, the program was entirely teacher-led and no additional support was provided. However, literature shows that school garden programs that for example provide an instructor, have support from stakeholders or provide lessons for teachers, could be more effective in changing (determinants of) vegetable consumption [20, 41, 42]. The importance of external support, especially at start-up of the school garden was also mentioned by teachers in a recent qualitative study on school gardens in Flanders (Belgium) [43]. Teachers were not obliged to implement all the lessons, which resulted in the fact that teachers implemented on average only four of the nine themes and most of the teachers indicated they could not teach the lessons as described in the manual. Teachers in the current study also found it difficult to implement the themes of the educational guide in their normal lessons, while integration in the curriculum is identified as one of the most important success factors of school gardening programs [20]. However, a previous study on school gardening in Flanders (Belgium), assessing implementation practices of existing school gardens, showed that also teachers with lots of experience with school gardening encounter difficulties to integrate working on the school garden in the curriculum [43]. Suggestions for integration of a school garden in the regular curriculum were indicated in the studies in the review of Ohly et al. [20], such as creating a fictitious business, relating songs and stories to the garden and measurements in mathematics. While the implementation by the teachers might be seen as a weakness in the scope of effects, it is of importance in the scope of later implementation and potential upscaling. Subsequently, for future school gardening programs in Belgium, it could be important to develop educational guides that have suggestions for lessons that can be linked to, for example, the governmental educational goals [43].

Another factor, not specifically related to the real-world program, possibly playing a major role in the lack of effects of the “Taste Garden” program is the fact that parents or the community were not involved in the project. Parents play a major role in the vegetable consumption of children [16, 24] and the involvement of parents in school-based nutrition programs is seen as important by teachers [43] and could potentially increase the effectiveness of the programs [16, 41, 44]. This was also concluded after the evaluation of the Royal Horticultural Society Campaign for School Gardening by Christian et al. (2014) [45]. There are several ways to involve parents, such as newsletters for parents, homework tasks for them and their children, parental evenings and involving them in maintenance of the garden during the school year or during holidays [43]. Also linking with the wider community is identified as a success factor of school garden programs [20]. Local organizations could for example be addressed for support and be involved in the maintenance of the school garden during holiday periods [43].

Another factor possibly causing the lack of effectiveness of the “Taste Garden” program is the fact that there was a very short program period (nine weeks), whilst longer and more intensive school-based programs are needed to change vegetable intake [46]. For example, most school garden programs effective in changing determinants of vegetable intake had an intensive program from 17 weeks up to an entire school year [34, 38, 39, 47, 48]. And although the studies of Morgan et al. (2010) and Jaenke et al. (2012), testing the effectiveness of a 10-week during nutrition education plus gardening intervention also showed changes in determinants of vegetable consumption [36, 37], future school garden programs should last long enough to be able to adequately address children’s vegetable consumption and its determinants. Also the period of the evaluation (October–December) was not ideal, as this is the fall season in Belgium and it is therefore very difficult to grow many vegetables in the school garden. This could have made it difficult to make the link between the lessons of the educational guide and the hands-on learning in the school garden planter. And last, the starter pack only contained one garden planter, which is rather limited to involve many children in the school garden. The majority of the children indicated that they wanted to work more in the school garden in the future, but this could possibly be difficult due to the small size of the school garden. It also makes it difficult to have a big harvest, which makes it difficult to work with the vegetables in class or use them for school meals. The fact that school garden planters are too small was also mentioned in previous research [43]. Schools could increase the size of their school garden by placing several garden planters. Local organizations could play a role in this as they could donate extra materials (e.g. soil, wood for the creation of new garden planters, seeds…) and community could also help in the expansion of school gardens by providing space for gardening nearby the school.

Even though the effectiveness of the “Taste Garden” program was very limited, and adaptations could be needed to increase effectiveness on vegetable consumption and its determinants, the program was already attractive to teachers and children and could potentially be beneficial for academic performance [49].

### Limitations

To our knowledge, this is the first study to integrate quality of implementation in the effect evaluation of a school garden project. However, this study has several limitations. First, there was a short evaluation period, without long-term follow-up. This makes it less likely to observe changes in (determinants of) vegetable consumption, as it is known that changing health behaviour, especially in children, is difficult and takes a substantial amount of time [50, 51]. Second, generalizability is limited as the project was only implemented in the urban region of Ghent (Flanders, Belgium). Third, the program was implemented by a local organization (i.e. Logo Gezond+) which resulted in the non-random assignment of schools to the intervention and control group, which could have caused selection bias. Furthermore, as purposive sampling was used, the intervention and control group were not matched on social and economic features. However, only a trend towards a significant difference in socioeconomic status was found. Fourth, the full impact of the program could not be estimated, as three schools dropped out of the evaluation because of motivational reasons and four schools were included in the control group as they did not set up the program in time. Fifth, although the questionnaire used in the evaluation was based on a validated questionnaire, data were self-reported which increases the likelihood of social desirable answers. Sixth, the evaluation only focused on vegetable consumption and its determinants, while the educational guide focused on more health behaviours and the promotion of these behaviours in class. Therefore, it may be possible that there were significant effects in other health related behaviours (e.g. drinking of water, unhealthy snacking…).

## Conclusions

Although previous research found several positive effects of school garden programs on health behaviour and wellbeing, it can be concluded that the “Taste Garden” program, at least in its current format and as delivered here, is not effective in increasing vegetable consumption and its determinants in primary schoolchildren and adaptations are needed. The lack of effects could be explained by the real-world nature of the program, time constraints and the lack of involvement of parents and the community in the program. However, the program was positively perceived by the school teachers and the children. Longer intervention periods and follow-up periods are necessary to capture the full effectiveness of school garden programs.

## Supporting information

S1 Table Content and answer categories of determinants. (DOCX)
