# Supplementary material for: Effect and process evaluation of a real-world school garden program on vegetable consumption and its determinants in primary schoolchildren
Source: PLoS One. 2019 Mar 21;14(3):e0214320. doi: 10.1371/journal.pone.0214320 (PMC6428286; doi:10.1371/journal.pone.0214320)
Supplement: S1 Table — (DOCX) [file pone.0214320.s001.docx]

| **Psychosocial correlate** | **Content** | **Answer categories** | **Cronbach’s Alpha** |
| --- | --- | --- | --- |
| Awareness | Do you think you eat many or few vegetables? | 5-point scale:   - Very few - Few - Not much, not few - Much - Very much | / |
| Knowledge | How many vegetable do you think you should eat each day to eat healthy? | 6 answering options:   - None - 1-3 tablespoons/day - 4-6 tablespoons/day - 7-9 tablespoons/day - 10-12 tablespoons/day - > 12 tablespoons/day   🡪 dummy coded:   - None – 4-6 tablespoons/day - 7-9 tablespoons/day - > 12 tablespoons/day | / |
| Social influence – social norm | Do you think you eat more or less vegetables than your peers? | 5-point scale:   - Much less - A little less - As much - A little more - Much more | / |
| Social influence – parental influence | - Do your parents demand that you eat vegetables every day? - My mother encourages me to eat vegetables every day - My father encourages me to eat vegetables every day | 5-point scale:   - Never - Seldom - Sometimes - Yes, almost every day - Yes, always   5-point scale:   - Totally disagree - Disagree - No opinion - Agree - Totally agree | 0.77 |
| Self-efficacy | - It is difficult for me to eat vegetables every day - If I decide to eat vegetables every day, I can - Eating vegetables every day is a habit for me | 5-point scale:   - Totally disagree - Disagree - No opinion - Agree - Totally agree | 0.78 |
| Attitude | - If I eat vegetables every day, I feel good - If I eat vegetables every day, I have more energy - I love to eat vegetables every day - Vegetables taste good | 5-point scale:   - Totally disagree - Disagree - No opinion - Agree - Totally agree | 0.84 |
